# Supplementary material for: The DNA adenine methylase of Salmonella Enteritidis promotes their intracellular replication by inhibiting arachidonic acid metabolism pathway in macrophages
Source: Front Microbiol. 2023 Mar 2;14:1080851. doi: 10.3389/fmicb.2023.1080851 (PMC10018194; doi:10.3389/fmicb.2023.1080851)
Supplement: Supplementary file 2 [file Data_Sheet_2.zip › S1 Appendix. Non-targeted metabolomics raw data/4.MetDiffAnalysis/C50336_WT.vs.control/C50336_WT.vs.control_pos_heatmap2.pdf]

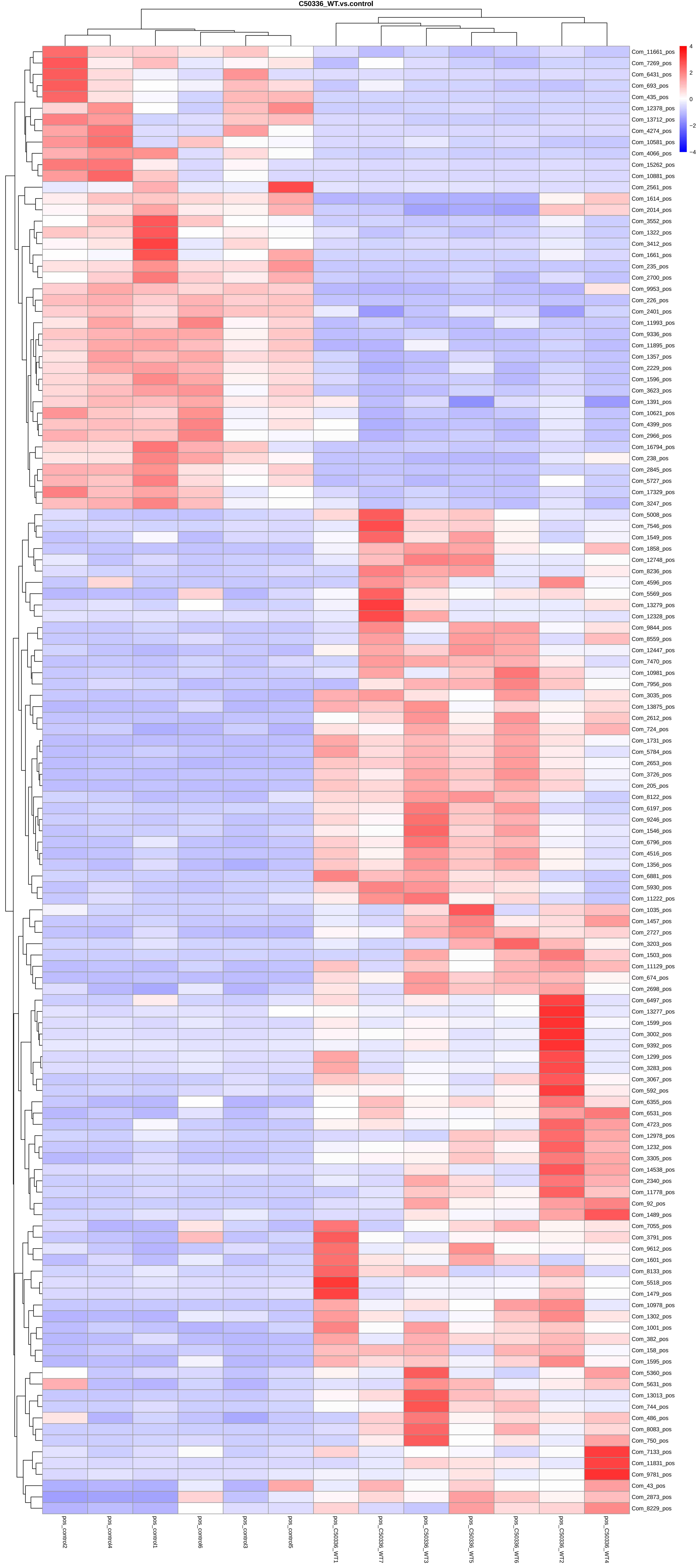
C50336_WT.vs.control
4
2
0
−2
−4
Com_11661_pos
Com_7269_pos
Com_6431_pos
Com_693_pos
Com_435_pos
Com_12378_pos
Com_13712_pos
Com_4274_pos
Com_10581_pos
Com_4066_pos
Com_15262_pos
Com_10881_pos
Com_2561_pos
Com_1614_pos
Com_2014_pos
Com_3552_pos
Com_1322_pos
Com_3412_pos
Com_1661_pos
Com_235_pos
Com_2700_pos
Com_9953_pos
Com_226_pos
Com_2401_pos
Com_11993_pos
Com_9336_pos
Com_11895_pos
Com_1357_pos
Com_2229_pos
Com_1596_pos
Com_3623_pos
Com_1391_pos
Com_10621_pos
Com_4399_pos
Com_2966_pos
Com_16794_pos
Com_238_pos
Com_2845_pos
Com_5727_pos
Com_17329_pos
Com_3247_pos
Com_5008_pos
Com_7546_pos
Com_1549_pos
Com_1858_pos
Com_12748_pos
Com_8236_pos
Com_4596_pos
Com_5569_pos
Com_13279_pos
Com_12328_pos
Com_9844_pos
Com_8559_pos
Com_12447_pos
Com_7470_pos
Com_10981_pos
Com_7956_pos
Com_3035_pos
Com_13875_pos
Com_2612_pos
Com_724_pos
Com_1731_pos
Com_5784_pos
Com_2653_pos
Com_3726_pos
Com_205_pos
Com_8122_pos
Com_6197_pos
Com_9246_pos
Com_1546_pos
Com_6796_pos
Com_4516_pos
Com_1356_pos
Com_6881_pos
Com_5930_pos
Com_11222_pos
Com_1035_pos
Com_1457_pos
Com_2727_pos
Com_3203_pos
Com_1503_pos
Com_11129_pos
Com_674_pos
Com_2698_pos
Com_6497_pos
Com_13277_pos
Com_1599_pos
Com_3002_pos
Com_9392_pos
Com_1299_pos
Com_3283_pos
Com_3067_pos
Com_592_pos
Com_6355_pos
Com_6531_pos
Com_4723_pos
Com_12978_pos
Com_1232_pos
Com_3305_pos
Com_14538_pos
Com_2340_pos
Com_11778_pos
Com_92_pos
Com_1489_pos
Com_7055_pos
Com_3791_pos
Com_9612_pos
Com_1601_pos
Com_8133_pos
Com_5518_pos
Com_1479_pos
Com_10978_pos
Com_1302_pos
Com_1001_pos
Com_382_pos
Com_158_pos
Com_1595_pos
Com_5360_pos
Com_5631_pos
Com_13013_pos
Com_744_pos
Com_486_pos
Com_8083_pos
Com_750_pos
Com_7133_pos
Com_11831_pos
Com_9781_pos
Com_43_pos
Com_2873_pos
Com_8229_pos
pos_control2
pos_control4
pos_control1
pos_control6
pos_control3
pos_control5
pos_C50336_WT1
pos_C50336_WT7
pos_C50336_WT3
pos_C50336_WT5
pos_C50336_WT6
pos_C50336_WT2
pos_C50336_WT4
